# Exploration of the relationship between Graves’ eye disease and type 2 diabetes based on biomarkers

**DOI:** 10.1007/s42000-025-00674-y

**Published:** 2025-05-31

**Authors:** Yu Guan, Meng Fan, Xiaolin Ren, Siyuan Zhang, Chun Cao, Jingbing Lan, Qiongfang Cao, Tiecheng Zhang, Fan Xu, Tao Zhang

**Affiliations:** 1 Department of Ophthalmology, The Second Affiliated Hospital of Chengdu Medical College, Nuclear Industry 416 Hospital, Chengdu, 610051 Sichuan China; 2 Department of Pathology and Pathophysiology, School of Basic Medical Science, Chengdu Medical College, Chengdu, 610500 Sichuan China; 3 Department of Biochemistry and Molecular Biology, School of Biological Sciences and Technology, Chengdu Medical College, Chengdu, 610500 Sichuan China; 4 Department of Biomedical and Experimental Teaching Demonstration Center, School of Bioscience and Technology, Chengdu Medical College, Chengdu, 610500 China; 5 Department of Evidence-Based Medicine and Social Medicine, School of Public Health, Chengdu Medical College, Chengdu, 610500 Sichuan China; 6 Sichuan Provincial Key Laboratory of Philosophy and Social Sciences for Intelligent Medical Care and Elderly Health Management, Chengdu Medical College, Chengdu, 610500 Sichuan China; 7 Department of Gastrointestinal Surgery, The Second Affiliated Hospital of Chengdu Medical College, China Nation Nuclear Corporation 416 Hospital, Chengdu Medical College, Chengdu, 610051 Sichuan China

**Keywords:** Type 2 diabetes, Graves’ Ophthalmopathy, Biomarker levels

## Abstract

**Purpose:**

The development of Graves’ ophthalmopathy (GO) is silent and can be accompanied by type 2 diabetes (T2D). However, the early diagnosis of these two conditions remains difficult.

**Methods:**

We evaluated a total of 123 patients with T2D and GO and 128 patients with GO treated in our hospital from 01 May 2016 to 31 May 2022. We determined the levels of several biomarkers and developed a regression model to evaluate the diagnostic efficacy of these biomarkers.

**Results:**

Univariate analysis showed that age and thyroid-stimulating hormone (TSH), free triiodothyronine (FT3), free thyroxine (FT4), glycated haemoglobin (HbA1c), and fasting glucose levels were significantly different in the GO-T2D group compared with the GO group. In the multivariate logistic regression analysis, FT3 and FT4 levels lost their statistical significance when the other factors remained unchanged. Older age and higher TSH, HbA1c, and glucose levels were associated with an increased likelihood of having GO-T2DM. The regression model for diagnosing GO and GO-T2D presented an R^2^ of 0.70, a sensitivity of 87.80%, a specificity of 93.75%, and an area under the receiver operating characteristic (ROC) curve of 0.97.

**Conclusion:**

Age and TSH, HbA1c, and glucose levels are effective predictors of GO and GO-T2D. Therefore, routine examination of these biomarkers in patients with GO could help to diagnose T2D early, thus allowing early treatment and a better prognosis.

## Introduction

Graves’ ophthalmology (GO) is an autoimmune disease and the main extrathyroid manifestation of Graves’ disease [1]. The most common clinical manifestations include periorbital oedema, conjunctival oedema, exophthalmos, diplopia, corneal ulcer, and upper eyelid retraction. In some severe cases, optic nerve compression may lead to vision loss [2]. This disease has a negative impact on quality of life [3–5] and creates a considerable public health burden in terms of both direct and indirect costs [3]. Therefore, early prevention and diagnosis are important. The common risk factors for GO include smoking, thyroid dysfunction, high serum thyroid-stimulating hormone (TSH) receptor antibody levels, radioactive iodine (RAI) therapy, and hypercholesterolaemia [1]. The diagnostic criteria mainly include abnormal thyroid function or thyroid-associated antibodies [6].

Type 2 diabetes (T2D) is the most common endocrine disease, causing high morbidity and mortality [7]. The latest epidemiology report disclosed that the proportion of the population with T2D is increasing rapidly, especially among younger people [8]. It is expected to affect nearly 1 in 3 people in the United States alone by 2050 [9]. T2D can lead to microvascular and macrovascular complications, causing profound psychological and physical distress to patients and caregivers and placing a huge burden on the healthcare system [10].

T2D and GO are common endocrine system diseases that are closely related, while T2D can be considered a risk factor for GO in patients with Graves’ disease or autoimmune thyroid disease [11]. The presence of T2D in patients with thyroid eye disease can be a predictive factor for the onset, progression, and severity of the disease [12]. In addition, thyroid hormones can increase blood glucose levels by regulating the serum levels of insulin and other hormones. Patients with T2D have a higher risk of optic neuropathy and corneal decompensation. Underlying T2D-related vascular disease may exacerbate this condition [13, 14].

However, there are few studies on patients with GO and T2D. It is unknown whether chronic unstable hyperglycaemia can affect the levels of thyroid hormones and other relevant laboratory indicators, thus further aggravating the clinical symptoms of GO. For example, the autoimmune inflammatory state of GO patients interferes with the normal secretion and action of thyroid hormones, affecting glucose-related hormones and signalling pathways [15]; The hyperglycaemia and metabolic disorders caused by T2D may further aggravate the immune inflammatory response [16], forming a vicious cycle. It is thus of note that thyroid function and blood glucose indicators can indicate this process [17] and. therefore, can be used as potential biomarkers for early diagnosis of GO-T2D.

Although many studies have revealed a link between thyroid dysfunction and diabetes, most of them have focused on the association between thyroid diseases and diabetes, such as the relationship between general hypothyroidism and diabetes [18–20]. To our best knowledge, few studies have disclosed the accurate early diagnostic indicators and effective prediction models. Moreover, the relationship between GO and T2D remains unclear. The aim of this study has therefore been to investigate the differences in thyroid hormones, related thyroid autoantibodies, and other related biochemical indicators in patients with GO and T2D compared with patients with GO. The results provide a theoretical basis for strategies to manage the early diagnosis and treatment of GO-T2D.

## Methods

### Participants

The study included 416 patients treated in an Eye Department from 2016 to 2022. Of these patients, 251 participated in the study, 128 diagnosed with GO and 123 patients diagnosed with GO-T2D. The inclusion criteria were as follows: a confirmed diagnosis of Graves’ disease accompanied by varying degrees of ophthalmopathic manifestations such as eyelid retraction, exophthalmos, conjunctival hyperaemia, and oedema and diplopia; a diagnosis of T2D meeting the diagnostic criteria established by the World Health Organization (WHO) or the American Diabetes Association (ADA); aged between 18 and 85 years old among male or female; and voluntary participation and a signed informed consent form. The exclusion criteria were as follows: patients with prediabetes and/or the presence of other types of thyroid or autoimmune diseases; recent (within the past 3 months) use of medication or surgical treatment that affects thyroid function or blood glucose levels (including but not limited to antithyroid drugs, insulin, metformin, etc.); coexistence of severe heart, liver, or kidney dysfunction; pregnant or lactating women; and declining to participate in the study or inability to complete all necessary examinations required for the study.

### Laboratory examinations

Serum TSH, free triiodothyronine (FT3), and free thyroxine (FT4) levels were measured using sensitive immunoassays, these levels reflecting pituitary regulation of thyroid function. Glycated haemoglobin (HbA1c) levels were assessed using either high-performance liquid chromatography (HPLC) or immunoturbidimetry, these levels reflecting average blood glucose levels over the past 2–3 months. TgAb and TPOAb levels were quantified using either the electrochemiluminescence immunoassay (ECLIA) or the immunoradiometric assay (IRMA) to evaluate the autoimmune status of the thyroid gland. High-sensitivity C-reactive protein (hs-CRP) levels were determined via an ultrasensitive turbidimetric assay, the latter serving as a sensitive marker of inflammation. Blood glucose levels were measured using the glucose oxidase method. Finally, experienced ophthalmologists and endocrinologists collaborated to conduct a clinical evaluation of GO, documenting the eye disease grade and symptom severity.

### Quality control

To minimise testing errors, all laboratory assays were conducted in a single laboratory, adhering to standardised operating procedures and quality control measures. Furthermore, the study personnel underwent uniform training to ensure consistency and accuracy in clinical assessments.

### Statistical analysis

All data were analysed using Stata 18.0. Continuous variables with normal distribution were expressed as mean ± standard deviation. The chi-square test or t-test for univariate analysis was used to examine the associations of TSH, FT3, FT4, HbA1c, TgAb, TPOAb, hs-CRP, and GLU levels with GO-T2D. Binary logistic regression was employed for multivariate analysis, using the K-fold class toolbox to implement K-fold cross-validation.The outcome variable in the binary logistic regression model determined whether the patient had Graves’ ophthalmopathy complicated by type 2 diabetes (GO-T2D) or not. A P-value < 0.05 was deemed to be statistically significant. Due to the small proportion of missing values, we used the list deletion method for missing values.

## Results

### Characteristics of the GO and GO-T2D groups

In total, 251 patients were included in this study, 128 with GO and 123 with GO-T2D. Table 1 shows the sex, age, and levels of the clinical parameters in the GO and GO-T2D groups. There was a significant difference in HbA1c level between the GO group (7.34 ± 1.26%) and the GO-T2D group (5.75 ± 0.40%; P < 0.001), indicating that HbA1c is a useful marker. Fasting glucose was significantly elevated in the GO-T2D group (7.83 ± 2.33 mmol/L) compared with the GO group (5.33 ± 0.78 mmol/L; P < 0.001), highlighting hyperglycaemia as a crucial characteristic of patients with GO-T2D. Age and TSH, FT3, FT4, HbA1c, and fasting glucose levels were the primary distinguishing factors between the GO and GO-T2D groups, whereas TgAb, TPOAb, and hs-CRP levels did not differ between the groups.

**Table 1:** Comparative analysis of the clinical parameters of patients with Graves’ ophthalmology (GO) and Graves’ ophthalmology and type 2 diabetes (GO-T2D)

Item	Category	GO (n = 128)	GO-T2D (n = 123)	χ^2^/t	P
Sex	Male, n (%)	54 (42.18)	47 (38.21)	0.412	0.521
	Female, n (%)	74 (57.82)	76 (61.78)		
Age	< 50 years old, n (%)	80 (62.50)	25 (20.32)	45.851	< 0.001
	≥ 50 years old, n (%)	48 (37.50)	98 (79.68)		
TSH(mIU/mL)	-	0.18 ± 0.73	4.08 ± 9.57	40.779	< 0.001
FT4(nmol/L)	-	25.57 ± 15.11	18.51 ± 8.30	18.015	< 0.001
FT3(pmol/L)	-	8.07 ± 4.88	5.26 ± 2.87	20.614	< 0.001
HbA1c(%)	-	5.75 ± 0.40	7.34 ± 1.26	58.304	< 0.001
TgAb(IU/mL)	-	551.38 ± 1359.00	330.38 ± 922.96	13.457	0.135
TPOAb(IU/mL)	-	119.78 ± 178.45	135.26 ± 184.92	1.358	0.500
hs-CRP(mg/L)	-	2.03 ± 4.36	2.05 ± 2.41	0.761	0.976
GLU(mmol/L)	-	5.33 ± 0.78	7.83 ± 2.33	49.501	< 0.001

*Abbreviation:*TSH,thyroid-stimulating hormone; FT4, free thyroxine; FT3, free triiodothyronine; HbA1c, glycated hemoglobin A1c; TgAb,thyroglobulin antibody; TPOAb,thyroid peroxidase antibody; hs-CRP, high-sensitivity C-reactive protein; GLU, glucose

Footnote: The chi-square test (test) was used to compare the differences between groups of categorical variables (such as gender and age groups). The t-test was used to compare the differences between groups of continuous variables (such as TSH, FT4, FT3, HbA1c, TgAb, TPOAb, hs-CRP, and GLU). All continuous variables were presented as (mean ± SD).

### GO and GO-T2DM group differences

**Fig. 1:**
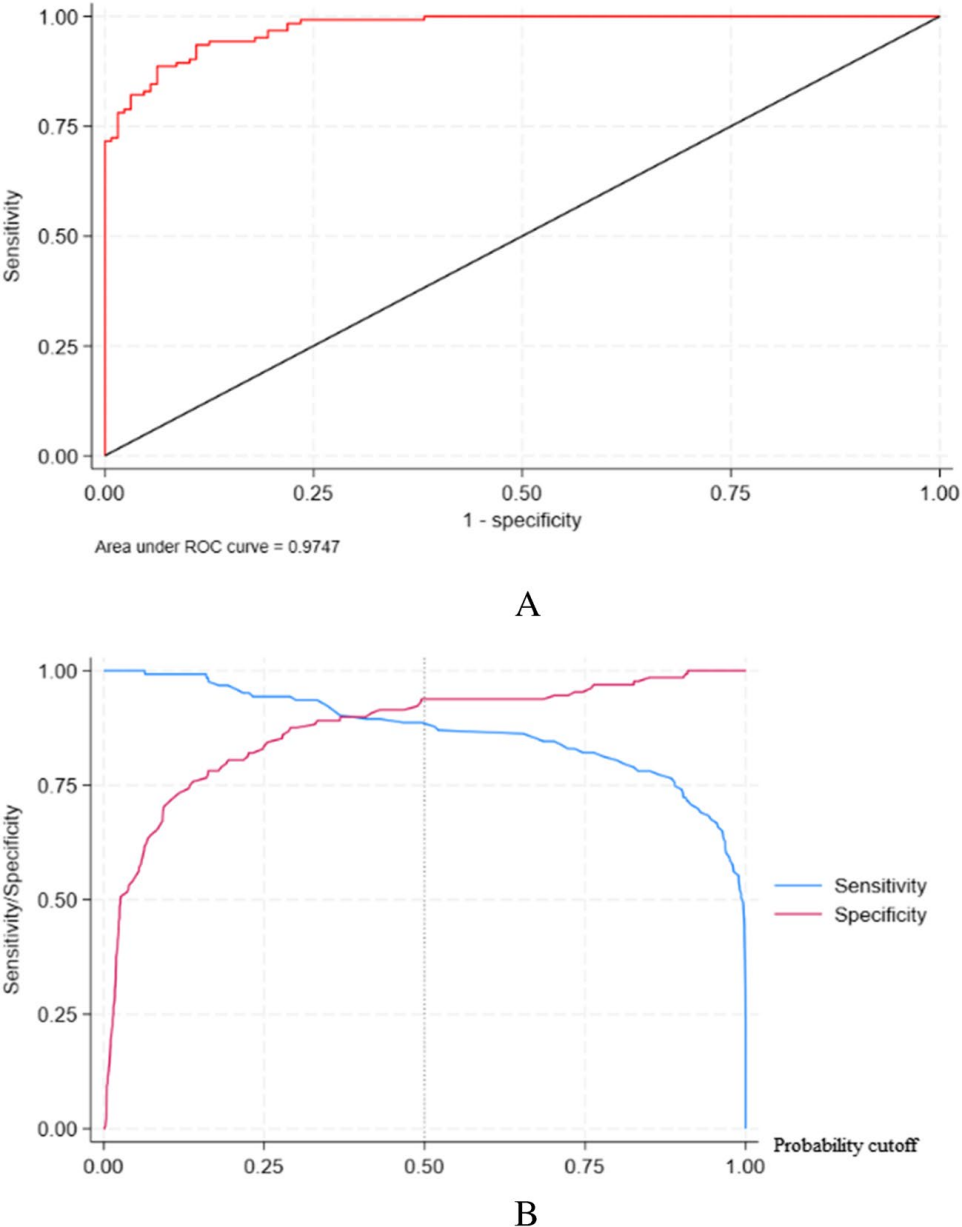
**A** ROC curve of the regression model **B** Predictive probability showing the sensitivity and specificity segmentation plots

We performed regression analysis with the presence or absence of GO complicated by T2D as the dependent variable, and age group (categorised as < 50 and ≥ 50 years) and TSH, FT4, FT3, HbA1c, and GLU levels as the independent variables. Based on binary logistic regression for multivariate analysis, age group and the TSH, HbA1c, and GLU levels were significant predictors of GO-T2D in this population, with older age and higher TSH, HbA1c and GLU levels associated with an increased likelihood of having GO-T2D (Table 2). The regression model had an R^2^ 0.70 value. It correctly classified 91.63% of the patients with GO-T2D (Table 3), the area under the receiver operating characteristic (ROC) curve being 0.97 (Fig. 1).

**Table 2:** Logistic regression analysis of factors associated with Graves’ ophthalmology and type 2 diabetes (GO-T2D)

Item	ORs	95%CI	P
Age group	6.055	(1.885, 19.445)	0.002
TSH	2.533	(1.563, 4.105)	< 0.01
FT3	0.927	(0.713, 1.204)	0.567
FT4	1.008	(0.930, 1.092)	0.85
HbA1c	11.842	(3.920, 35.774)	< 0.01
GLU	2.465	(1.485, 4.091)	< 0.01
cons	6.055	(1.885, 19.445)	< 0.01

*Abbrevition:*TSH, thyroid-stimulating hormone; FT4, free thyroxine; FT3, free triiodothyronine; HbA1c, glycated hemoglobin A1c; GLU, glucose

**Table 3:** Predictive value of the regression model

Predictor	Rate
Sensitivity	87.80%
Specificity	93.75%
Positive predictive value	93.10%
Negative predictive value	88.89%
Correctly classified	90.84%

### Collinearity diagnostics

FT3 and FT4 did not present any statistical significance in the multivariate analysis, while collinearity diagnosis found the following key results only when TSH served as the dependent variable, namely, 93% of FT3 and FT4 in the third dimension, respectively. The condition index was 10.351, suggesting mild collinearity of both. There was no collinearity between TSH and FT3/FT4 (all conditions were < 4) (Table 4).

**Table 4:** Collinearity diagnostics summary

Dependent variable	Max condition index	FT3 variance proportion (Dimension 3)	FT4 variance proportion (Dimension 3)	Collinearity conclusion
FT3	3.996	0.92	N/A	No collinearity
FT4	3.702	N/A	0.91	No collinearity
TSH	10.351	0.93	0.97	Mild collinearity between FT3 and FT4

Footnotes:

Condition index: A threshold > 10 indicates potential collinearity

Variance proportion: High values (> 0.9) in the same dimension suggest shared variance

TSH analysis: FT3 and FT4 show mild collinearity when TSH is the dependent variable

FT3/FT4 analyses: No collinearity detected when FT3 or FT4 is the dependent variable

### K-fold cross-validation of the regression model

**Fig. 2:**
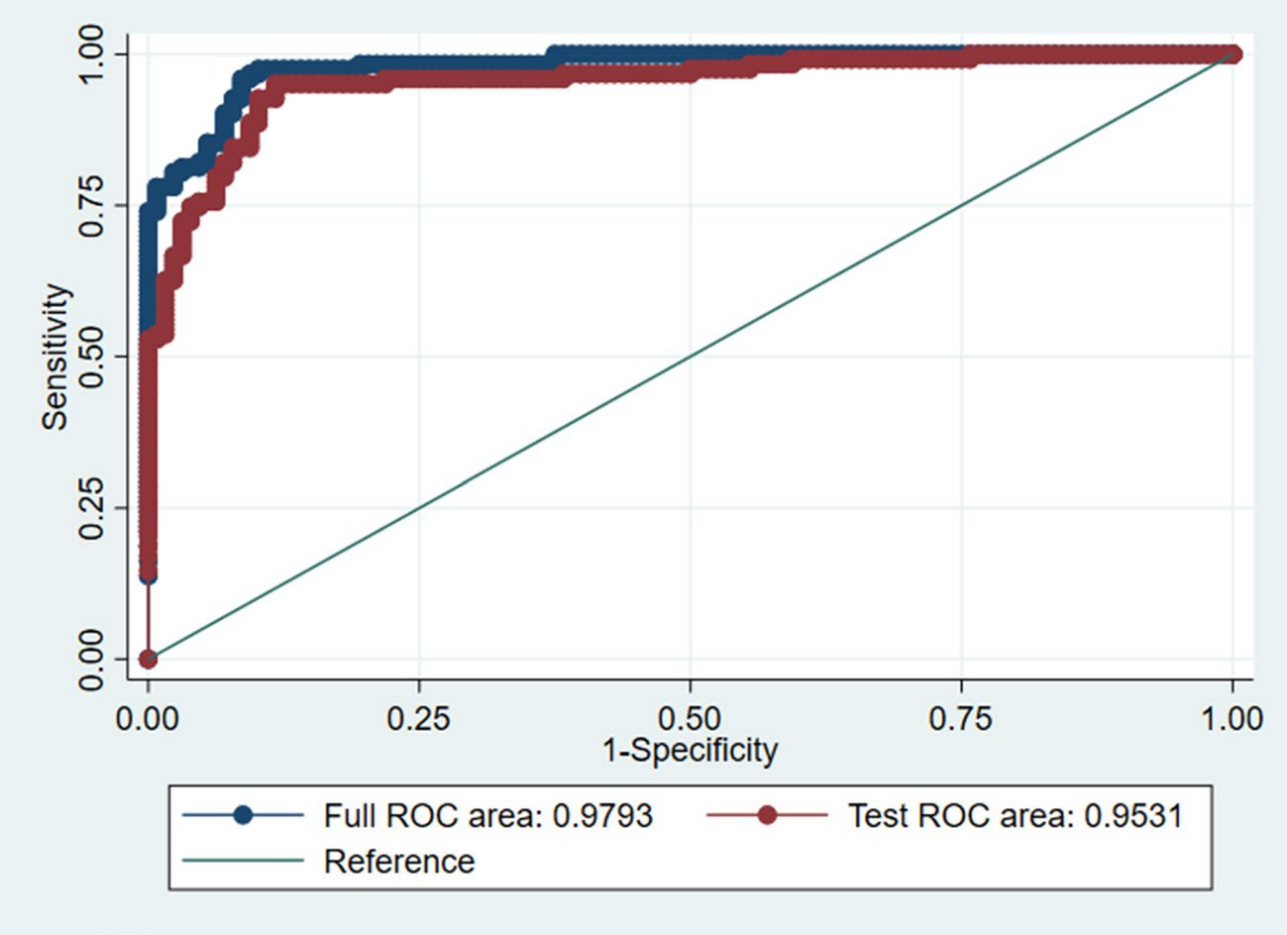
ROC curve after K-fold cross-validation

To address potential concerns regarding overfitting, we performed K-fold cross-validation of the regression model. After K-fold cross-validation, the ROC curve of the model on the test set, AUC value is 0.9531, indicating that the logistic regression model shows high classification accuracy and reliability based on both the whole data and the test set data (Fig. 2).

## Discussion

GO and T2D are common endocrine diseases with a common genetic immunological basis [21]. Due to immune system dysfunction and various genetic defects, the immune abnormalities of GO and T2D may exert synergistic effects precipitating the development of the disease [22]. In addition, patients with GO and T2D have several similar clinical manifestations that can lead to a missed diagnosis or a misdiagnosis [23]. Recently, researchers have uncovered the correlation between the severity of thyroid eye disease and T2D [12]. Epidemiological studies have confirmed that the incidence of abnormal thyroid function in patients with T2D is 2–3 times that of patients without T2D [24]. GO may not cause T2D, but it can aggravate this disease. Based on these facts, determining which biomarkers are potential indicators of GO-T2D could be important for early diagnosis and clinical decision-making.

We found that age and TSH, HbA1c, and fasting glucose levels were the main factors that distinguish between patients with GO-T2D and GO, while sex, thyroid autoantibodies, and inflammatory markers did not. We also observed that the older the age, the greater the risk of GO-T2D. Consistently, researchers have shown that age is a risk factor for GO [25] and T2D [26], these results therefore pointing to the need to improve risk assessment particularly among older patients with GO. There were significant differences between the GO-T2D and GO groups as concerns FT3 and FT4 levels in the univariate analysis, this being consistent with previous study results [27]. However, FT3 and FT4 levels failed to show diagnostic value in the regression prediction model, which may be due to the correlation between FT3, FT4, and TSH [28, 29]. Collinearity by linear diagnosis falsely increased the regression coefficient criteria of FT3 and FT4, which reduced the statistical significance. The significance of TSH was not affected due to its strong independence. After including TSH in the model as a covariable, the effects of FT3 and FT4 levels were not significant. In subsequent studies we intend to combine FT3 and FT4 into comprehensive indicators (such as by employing principal component analysis) in the analysis with TSH as the dependent variable. Sample size was increased and the model stability regularisation method (such as ridge regression) was used to optimise model stability.

TSH is a glycoprotein hormone produced by the anterior pituitary gland. It is the primary stimulus for thyroid hormone production by the thyroid gland. It also exerts growth effects on thyroid follicular cells, leading to goitre [30]. Based on previous studies, hypothyroidism (decreased TSH) is common in patients with diabetic peripheral neuropathy and correlates independently with its severity [31], which is also reflected in the correlation between TSH and diabetes. Based on the present study, it is therefore apparent that TSH may be used as a diagnostic biomarker for GO and GO-T2D, since the lower the TSH, the greater the risk of GO-T2D.

HbA1c is a key indicator for measurement of blood glucose level [32]. An elevated HbA1c level usually indicates an underlying abnormality in glucose metabolism. HbA1c is formed via the combination of haemoglobin and glucose in serum [33], which is closely related to the lifespan of red blood cells and the concentration of glucose in the blood [34]. It mainly reflects the level of blood glucose control over the past 2–3 months [35]. Elevated HbA1c levels also increase the release of inflammatory molecules, including CRP, interleukin (IL)−1β, IL-6, and IL-17 through chronic inflammation [36]. Many researchers have applied measurement of HbA1c levels to predict the risk of carotid atherosclerosis and metabolic syndrome-related fatty liver disease [37].

Taken together, immune dysfunction and inflammation are important factors in the development of the disease in GO patients. TSH is thought to regulate immune cell function and to participate in immune response process and inflammation microenvironment regulation. An increase of HbA1c induces a series of inflammatory reactions, damages blood vessels and tissues, and affects insulin signalling; meanwhile, abnormal glucose metabolism leads to impaired immune cell function and increased inflammatory mediators. The three interact together to promote the progress of GO and T2D.

Currently, TSH level is used to determine the treatment of GO [37] and to assess the severity of the disease [38]. However, the regression model revealed that age and TSH, HbA1c, and GLU levels are effective parameters to distinguish between GO and GO-T2D, with an area under the ROC curve of 97% and an accuracy of 91%. This model has the following advantages over traditional methods: it is more objective than clinical scoring systems (e. g., CAS) and more cost-effective than imaging models; and in the case of T2DM, it complements traditional glucose testing (e.g., fasting glucose and vulnerability to interference) and may enable early screening. T2D and GO share common biological mechanisms, including chronic inflammation, immune dysregulation, and oxidative stress. In GO, TSHR and IGF-1R antibodies drive inflammation, while in T2D, cytokines such as TNF-α and IL-6 exacerbate insulin resistance. Hyperglycaemia in T2D increases oxidative stress, promoting orbital fibroblast activation in GO. Adipokines (e.g., leptin) and genetic factors (e.g., CTLA-4) further link these conditions. Routine monitoring of TSH, HbA1c and glucose levels in GO patients is helpful for early diagnosis and treatment of T2D and improves prognosis. For patients with GO who are older (such as the significantly different age boundaries analysed in this study) and have abnormal TSH levels, blood glucose indicators should be closely monitored.

## Limitations

The current study has several limitations. First, since an effective cutoff value for diagnosis of GO-T2D has not yet been proposed, more research is required. Second, although some confounders were controlled in the study, there may still be unidentified or uncontrolled factors, such as patients’ genetic background, BMI, lifestyle (diet, exercise habits, and smoking), and other comorbidities, which may interfere with the relationship between biomarkers and GO-T2D and affect the accuracy of the study results. In addition, this study was based on cross-sectional data and lacked longitudinal follow-up to determine whether there is a causal relationship between the biomarker and GO-T2D: only an association could be found. Future studies need to conduct longitudinal follow-up to observe the time sequence of biomarker changes and disease occurrence and development, clarify the causal relationship, and further improve the understanding of GO and GO-T2D.

## Data Availability

All data will be made available upon reasonable request.
