# Correction: Exploration of the relationship between Graves’ eye disease and type 2 diabetes based on biomarkers

**DOI:** 10.1007/s42000-025-00684-w

**Published:** 2025-06-13

**Authors:** Yu Guan, Meng Fan, Xiaolin Ren, Siyuan Zhang, Chun Cao, Jingbing Lan, Qiongfang Cao, Tiecheng Zhang, Fan Xu, Tao Zhang

**Affiliations:** 1 Department of Ophthalmology, The Second Affiliated Hospital of Chengdu Medical College, Nuclear Industry 416 Hospital, Chengdu, 610051 Sichuan China; 2 Department of Pathology and Pathophysiology, School of Basic Medical Science, Chengdu Medical College, Chengdu, 610500 Sichuan China; 3 Department of Biochemistry and Molecular Biology, School of Biological Sciences and Technology, Chengdu Medical College, Chengdu, 610500 Sichuan China; 4 Department of Biomedical and Experimental Teaching Demonstration Center, School of Bioscience and Technology, Chengdu Medical College, Chengdu, 610500 China; 5 Department of Evidence-Based Medicine and Social Medicine, School of Public Health, Chengdu Medical College, Chengdu, 610500 Sichuan China; 6 Sichuan Provincial Key Laboratory of Philosophy and Social Sciences for Intelligent Medical Care and Elderly Health Management, Chengdu Medical College, Chengdu, 610500 Sichuan China; 7 Department of Gastrointestinal Surgery, The Second Affiliated Hospital of Chengdu Medical College, China Nation Nuclear Corporation 416 Hospital, Chengdu Medical College, Chengdu, 610051 Sichuan China

**Correction: Hormones**

10.1007/s42000-025-00674-y

In the original version of this article, the given and family names of Meng Fan were incorrectly structured.

The original article has been corrected.

